# Climate Change and Child Health Inequality: A Review of Reviews

**DOI:** 10.3390/ijerph182010896

**Published:** 2021-10-17

**Authors:** Emmanuelle Arpin, Karl Gauffin, Meghan Kerr, Anders Hjern, Angela Mashford-Pringle, Aluisio Barros, Luis Rajmil, Imti Choonara, Nicholas Spencer

**Affiliations:** 1 Canadian Center for Health Economics, University of Toronto, Toronto, ON M5T 3M6, Canada; emmanuelle.arpin@mail.utoronto.ca; 2 Centre for Health Equity Studies, Department of Public Health Sciences, Stockholm University, 10691 Stockholm, Sweden; anders.hjern@su.se; 3 Temerty Faculty of Medicine, University of Toronto, Toronto, ON M5S 1A8, Canada; meghan.kerr@mail.utoronto.ca; 4 Department of Medicine, Karolinska Institutet, 17177 Solna, Sweden; 5 Dalla Lana School of Public Health, University of Toronto, Toronto, ON M5T 3M7, Canada; angela.mashford.pringle@utoronto.ca; 6 Center for Epidemiological Research, Universidade Federal de Pelotas, Pelotas 96010-610, RS, Brazil; abarros@equidade.org; 7 Independent Researcher, Homer 22, 1rst 1, 08023 Barcelona, Spain; 12455lrr@comb.cat; 8 School of Medicine, University of Nottingham, Derby DE22 3DT, UK; imti.choonara@nottingham.ac.uk; 9 Warwick Medical School, University of Warwick, Coventry CV4 9JD, UK; n.j.spencer@warwick.ac.uk

**Keywords:** climate change, children, health inequality, scoping review, global health

## Abstract

There is growing evidence on the observed and expected consequences of climate change on population health worldwide. There is limited understanding of its consequences for child health inequalities, between and within countries. To examine these consequences and categorize the state of knowledge in this area, we conducted a review of reviews indexed in five databases (Medline, Embase, Web of Science, PsycInfo, Sociological Abstracts). Reviews that reported the effect of climate change on child health inequalities between low- and high-income children, within or between countries (high- vs low–middle-income countries; HICs and LMICs), were included. Twenty-three reviews, published between 2007 and January 2021, were included for full-text analyses. Using thematic synthesis, we identified strong descriptive, but limited quantitative, evidence that climate change exacerbates child health inequalities. Explanatory mechanisms relating climate change to child health inequalities were proposed in some reviews; for example, children in LMICs are more susceptible to the consequences of climate change than children in HICs due to limited structural and economic resources. Geographic and intergenerational inequalities emerged as additional themes from the review. Further research with an equity focus should address the effects of climate change on adolescents/youth, mental health and inequalities within countries.

## 1. Introduction

The uneven distribution of social and environmental factors on birth and early life give rise to avoidable child health inequalities [1]. Differences in child survival, health, development and well-being are stark between low- and middle-income countries (LMICs) and high-income countries (HICs) [2]. Many children in LMICs live in circumstances in which they are deprived of essential determinants of health such as clean air, adequate shelter, nutrition, safe water and sanitation [3], all of which contribute to the higher risk of adverse child health outcomes such as stunting secondary to malnutrition [4], acute respiratory illness [5], diarrheal disease [6] and vector-borne diseases such as malaria [7]. Despite improvement in child survival rates within these countries, children from poorer households remain disproportionately vulnerable: on average, the risk of dying before age 5 is twice as high for children born into the poorest households as it is for those born into the richest [3]. Inequalities within HICs exist as well with many children in low-income households experiencing high levels of air pollution [8], food insecurity [9] and poor housing conditions [10].

Climate change is an ongoing urgent global problem. The recently published sixth assessment Intergovernmental Panel on Climate Change (IPCC) [11] asserts that anthropogenic greenhouse gas emissions have been responsible for an increase in yearly average temperatures across the world currently estimated at 1.2 °C over pre-industrial temperature levels. Without mitigation, global temperature change will likely increase by 1.5 °C by 2030 and may increase 4.8 °C by 2100. Observable planetary changes due to climate change including glaciers melting, water levels rising, prolonged heat waves, floods, droughts and rainfall have accelerated in 2020–21 with uncontrolled wild fires in the west coast of North America, parts of Australia and southern Europe and unprecedented flooding in China and central Europe.

Global warming and its consequences are now accepted as a significant threat to global health and well-being, and children are known to be particularly vulnerable to its effects [12]. In 2009, Lancet Commission on Climate Change determined that climate change is the biggest global health threat of the 21st century [13]. The commission concluded that most health impacts will be adverse and will occur via direct exposures (e.g., heat waves, extreme weather events) but also by significantly impacting basic social determinants of health. The commission further identified certain populations as “vulnerable” to climate change such as the elderly, children, individuals with underlying health conditions and populations in LMICs. Focusing on children more specifically, a scoping review published after the end date of our search [14], identified the range of childhood conditions exacerbated by direct and indirect effects of climate change, for example, vector-, water- and food-borne infectious diseases and mental health problems.

We conducted a scoping review of published review articles with the aim of assessing the strength of evidence for the extent and mechanisms by which climate change and its consequences differentially impact children in social groups within countries and in poorer compared with richer countries and identify knowledge gaps. The main research questions of the review were: What is the current state of knowledge on the impact of climate change and its consequences on child health inequalities? What is the evidence that climate change exacerbates child health inequalities? Is the evidence reported in the reviews supported by quantitative data comparing the impact of climate change and its consequences on different social groups within countries and/or between countries? Are the mechanisms by which climate change and its consequences may exacerbate and/or generate child health inequalities addressed in the included reviews?

## 2. Materials and Methods

We conducted a scoping review, guided by the methodology outlined by Arksey and O’Malley [15], to examine the main research questions listed above. The population of interest is children aged 0–18 years, the key concepts are climate change and inequalities in child health and the contexts of interest are social groups within countries, low- and middle-income countries (LMICs) compared to high-income countries (HICs) and geographical locations.

The scoping review approach was favored as it allows researchers to, “identify, retrieve and summarize literature relevant to a particular topic for the purpose of identifying the key concepts underpinning a research area and the main sources and types of evidence available” [15] ( p. 14). In recent years there have been advancements in the methodology [16] and a rapid increase in its application due to its wide range of uses [17]. According to Arksey and O’Malley, two features of a scoping review contrast it from a systematic review. First, where systematic reviews have a well-defined question and may examine a particular type of study design, a scoping review addresses broader questions, allowing the researcher to identify and map key concepts of an area, and may include various study designs. Second, unlike a systematic review, a scoping review does not include a quality assessment of the included studies. Arksey and O’Malley further suggest that scoping reviews may have one of two purposes, either to serve as a first step towards a subsequent systematic review or research project, or it may be conceived as a method in its own right, to identify key concepts or gaps in the existing evidence and define the main sources of evidence.

Building on the scoping review approach, we chose to scope reviews rather than primary literature. The ”review of reviews” approach is efficient when aiming to capture how a concept is described in the literature and to map out emerging themes, rather than relying on primary literature. Examples of applications are in the examination of loneliness and social isolation interventions for older adults [18] and mental health promotion interventions [19].

In partnership with the Karolinska Institute in Stockholm, we led a review of peer-reviewed reviews indexed in five databases (Medline, Embase, Web of Science, PsycInfo, Proquest). The search was conducted from database start dates to 21 January 2021. Search terms used for all databases are shown in the supplementary files (Tables S1 and S2). We defined climate change in broad terms. Examples of search terms were “Climate Change”, “Greenhouse Effect”, “Hot Temperature”, “Natural disaster”, “Heat wave” and “Wildfire”. The population of interest was children and young people less than 18 years old which we defined according to the age definition of the UN Convention of the Child [20]. We considered a range of health conditions, including physical ailments, infectious diseases and mental health conditions. Mental health was defined broadly with the inclusion of disorders as well as psychological consequences of climate change. We defined inequalities in relation to specific indicators such as socioeconomic status, income, wealth, poverty, ethnicity and indigenous status within and between countries. There were no limits on years of publication nor languages.

The inclusion and exclusion criteria are shown in Table 1.

**Figure 1:**
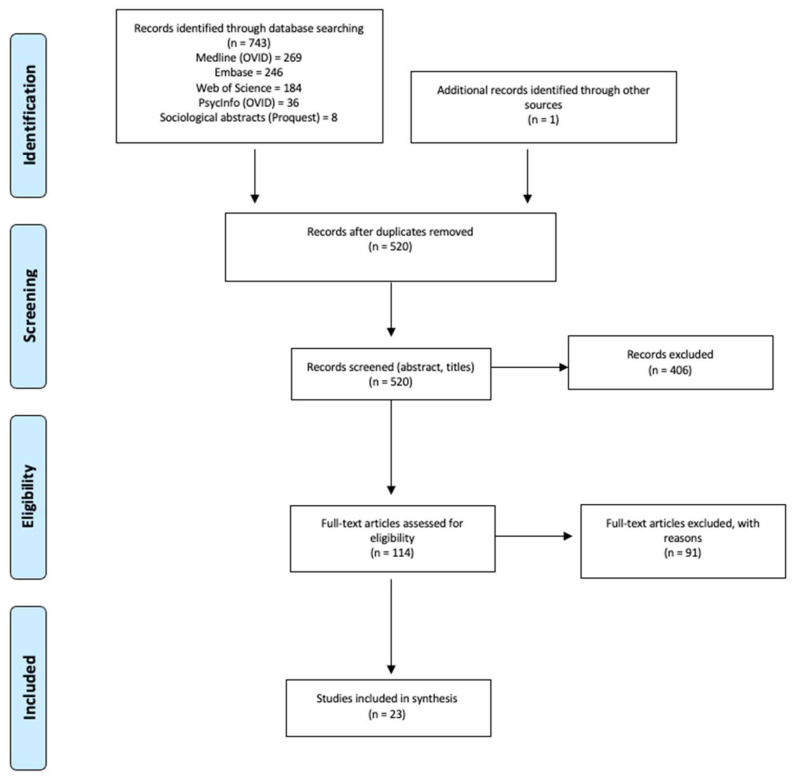
PRISMA Flow Diagram.

Our search strategy retrieved 743 reviews, with 520 reviews after deduplication. Though not retrieved in the initial search, one additional review article was included for its relevance to the topic and objective. Title and abstract evaluations rendered 114 reviews eligible for full-text analysis. Papers for full text analysis were allocated to groups of two researchers, each to undertake review and reach consensus. Disagreements were resolved by whole group decision. Following this, 91 articles were excluded leaving 23 reviews that were included for synthesis. The results of the review process are presented as a PRISMA flow diagram in Figure 1. In addition, we followed the charting approach described by Arksey and O’Malley and Levac et al. to synthesize results and comment on emerging themes.

The primary theme of interest in the scoping review was the association of climate change and child health inequalities reported in three specific dimensions; within country differences by social groups, between country differences (LMICs vs. HICs), and living in specific geographical locations. For within country inequalities, we sought evidence that reviews identified the impact of climate change on children in social groups by relative advantage versus disadvantage. For between country inequalities, we explored how reviews reported and explained the differential impact of climate change on child health in LMICs compared with HICs. Geographical location was explored as a dimension of inequality as children living in these areas were identified as at increased risk of adverse health outcomes resulting from climate change. In all these dimensions of inequality, we sought supporting quantitative and/or descriptive evidence of exacerbation of existing child health inequalities by climate change and evidence of the mechanisms by which climate change impacts child health inequalities. Definitions and descriptions of climate change and of child health in the reviews were explored as secondary themes.

## 3. Results

### 3.1. Characteristics of the Studies

Of the included reviews, four were systematic reviews (Table 2) [21,22,23,24], three were technical and commissioned reports (Table 3) [13,25,26] and 16 were narrative reviews, or opinion pieces with substantive literature reviews (Table 4) [27,28,29,30,31,32,33,34,35,36,37,38,39,40,41,42]. All reviews and reports were published in international peer-reviewed journals with the exception of the Assembly of First Nations Report which was included as the challenges faced by indigenous children and their families are under-represented in the literature. Sixteen reviews had a global focus incorporating LMICs and HICs [13,23,24,25,27,28,29,30,31,32,34,35,37,39,40,42], four reviews had a specific country focus (US [21], Canada [26,33], Cambodia [41]) and three reviews focused on grouped nations of a specific world region (LMICs [22,24], Sub-Saharan Africa, North America). Reviews were published from 2007 to 2020 and all were published in English.

### 3.2. Child Health Inequalities

#### 3.2.1. Within Country Inequalities

Within country inequalities in child health related to climate change were reported in 16 reviews [13,21,22,23,24,26,27,28,29,31,33,35,37,40,41,42]. All reviews included descriptive evidence and five included quantitative evidence [13,23,24,25,41]. Compared with more advantaged groups, children in poor, low income, low educated, socioeconomically marginalized households and those in indigenous and traditional societies, were identified as more likely to suffer consequences of climate change. Bennett and Friel [42] concluded that climate change acts as an amplifier of existing inequities with the result that the world’s poorest and socially-disadvantaged children will bear the greatest burden of climate change-related ill-health. Chersich et al. [23] reported that Korean women with both low education levels and low socioeconomic status had a 1.1-fold increased hazard ratio of preterm birth for each quartile increase in temperature compared with more socioeconomically advantaged women. Ahdoot and Pacheco [25] stated that the world’s poorest children are up to 10 times more likely to be affected by climate change associated weather disasters compared to children in higher-income families due to limited material and financial resources. Following floods in 2011, Cambodian children from households with poor sanitation and untreated drinking water as well as having a mother who lacked education experienced higher rates of diarrhea than households with treated water, soap and more highly educated mothers [41]. Lieber et al. [24] quantify the moderating effects of low socioeconomic status (−0.6) and high maternal education (+0.9) on childhood malnutrition although the exact metric used is not stated.

Four of the 13 included articles in Benevolenza and DeRigne’s systematic review [21] discussed the negative impact of climate change, specifically increased frequency and severity of hurricanes, on low-resourced/low-income parents and children. The authors found that children from low-income families and families led by single mothers are likely to suffer from more adverse mental, emotional and physical health problems than their more advantaged peers although, as the authors acknowledge, there was an absence, in all included studies, of statistics comparing disadvantaged with advantaged populations.

#### 3.2.2. Between Country Inequalities

Between country inequalities, comparing LMICs with HICs, were reported in 16 reviews [13,23,24,25,27,31,32,34,35,36,38,39,40]. All reported descriptive evidence and three reported quantitative evidence [13,23,25]. Ahdoot and Pacheco [25] reported climate change will increase the diarrheal disease burden leading to an estimated 48,000 additional deaths attributable to diarrheal disease in Asia and sub-Saharan Africa by 2030. In African populations, loss of healthy life years is predicted to be 500 times greater than in European populations as a result of climate change with devastating effects on African children’s life chances [13]. Anderko et al. [38] identify food insecurity as a consequence of extreme weather events and changes in temperature and precipitation patterns damaging and destroying crops, leading to increased malnutrition especially in sub-Saharan Africa and South Asia. Philipsborn and Chan [35], based on the projected increase in diarrhea, malaria and nutritional deficiencies due to climate change, predict that LMICs will experience an increased burden of avoidable death among under 5-year-old children. Rylander et al. [36] predict an increase in the risk of pregnancy complications, preterm delivery and low birthweight due to the expected increase in incidence of malaria, dengue fever and schistosomiasis among pregnant women in LMICs due to climate change.

#### 3.2.3. Geographic Inequality

The differential impact of climate change on child health was also defined along geographic lines [28,30,31,41]. Kistin et al. [30] discussed an increasing risk of flooding and water scarcity as a result of increasing temperatures, earlier thawing of snowpack and glaciers melting among communities in mountainous regions. Levy and Patz [31] highlighted climate change threats to coastal regions from rising sea levels. Another study found that children living in usually cooler climates will begin to become more vulnerable to climate change related illnesses and infections, such as Lyme disease and Dengue [28].

#### 3.2.4. Intergenerational Inequity

An additional dimension of inequality affecting children was identified by Ebi and Paulson [28] who defined ‘intergenerational inequity’, where children are considered to be a particularly disadvantaged population, not only because of physiological and developmental vulnerability, but also because their higher likelihood of experiencing severe effects of climate change in the future. McMichael [32] suggests that children will inevitably be exposed to adverse health effects for a greater portion of their lives than adults and will suffer the consequences of actions in which they have played little or no part. As such, the particular vulnerability of children to the detrimental effects of climate change may be regarded, not only as a result of young age, but also as a cohort effect.

### 3.3. Explanatory Mechanisms

Bennett and Friel [42] demonstrate how differential access to protective factors determined by family financial and social resources ensure that the child from a high-income family is less likely to be exposed to the worst effects of heat, flooding and other consequences of extreme weather events. As vector-borne diseases become more prevalent as a result of climate change, poor and disadvantaged children are at increased risk due to lifelong poverty, persistent malnutrition, insanitary living conditions and lack of preventive health measures.

The increased impact of climate change on child health in LMICs is attributed in the reviews to factors such as low levels of maternal education and poor water sanitation, poor infrastructure (e.g., air quality control, pollution control), weak economy, as well as poor political leadership and democratic institutions. Some reviews characterized LMICs as having a “double burden” of climate change based on their higher exposure to extreme weather events and on their limited capacity to mitigate the negative effects of climate change due to scarce structural economic and political resources [13,22,32,35,39].

A number of reviews postulated ‘direct’ effects and ‘indirect’ effects of climate change on child health [24,25,27,32]. Direct effects are the immediate impacts that climate change will have on children, while indirect effects are the impacts that climate change will have on important social determinants of health for children with downstream effects on child health. For example, the reduction in water supply following a severe weather event due to climate change will impact the food supply which can lead to childhood malnutrition and stunting over time. McMichael [32] further suggests that there are diffuse or ‘tertiary’ effects of climate change, which are further downstream effects such as physical displacement, impaired recreational facilities and limited opportunities for children.

### 3.4. Climate Change

We identified the variation in how climate change was defined across included reviews as a secondary theme. Although definitions varied, all are recognized as climate change and/or its consequences in the IPCC report [11]. The majority of reviews (14 reviews) discussed climate change as a gradual increase in planetary ambient temperature leading to increases in extreme weather events and summarized their respective health effects [13,26,27,28,29,31,32,34,35,36,37,38,39,40]. Few reviews explicitly defined and examined a specific feature of climate change. Nine reviews considered discrete weather events that are associated with climate change: increasing hurricane severity and frequency [21], increasing air pollution [25], weather events impacting droughts, floods, rainfalls [22,24,30,31,41,42], and heat waves [23,31,42]. However, the relationship between specific weather events and climate change was not always clear and at times minimized in some of the reviews [21,28].

### 3.5. Childhood and Child Health

An additional secondary theme to emerge from the reviews was how childhood and child health were defined. Most reviews included all ages from birth to 18, while others focused on children less than 5 years of age [22,34,36] or the antenatal and/or perinatal periods [23,36,37,38,40]. Reviews emphasized children’s vulnerability to the health effects of climate change, compared to adults, due to their incomplete physiological and cognitive development, as well as their dependence on parents and/or caregivers [29,36,37,38] and their higher exposure to air, food and water per unit body weight [27,39].

Multiple adverse health conditions were discussed in the reviews: malnutrition [24,30,31,37,38,39], malnutrition with associated stunting and increased susceptibility to infection [22,32], vector-borne diseases [13,25,28,29,30,31,32,35,37], respiratory diseases [13,26,28,31,32,37,39], diarrhea mainly caused by water-borne diseases [13,28,30,41], perinatal health such as gestational age and birth weight [23,36,38,40], and mortality [28,35]. Only three reviews explicitly considered child mental health and cognitive development [21,27,40], where anxiety, post-traumatic stress disorder and learning disruptions due to displacements were studied. Finally, two reviews focused on indigenous children in Canada considered dimensions of spiritual health, which significantly broadened the definition of child health compared to other reviews [26,33].

## 4. Discussion

**Figure 2:**
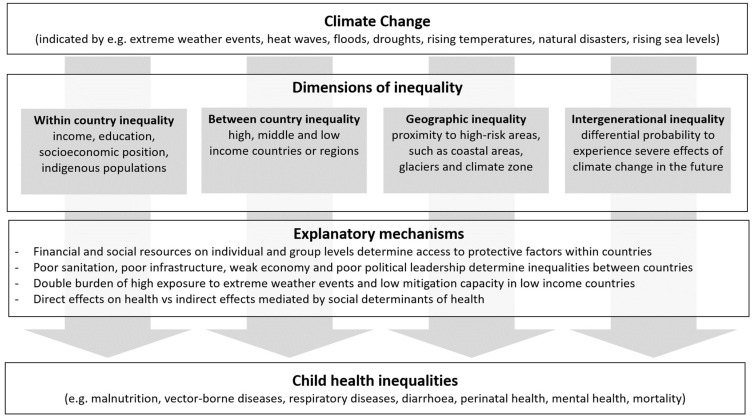
Relationship between climate change and child health inequalities.

This scoping review of reviews identifies the current level of knowledge and gaps in the literature on the effect of climate change on child health inequalities. Figure 2 presents a graphical representation of the findings of the scoping review.

While inequalities in child health within and/or between countries or geographical regions are touched upon in all the included reviews, few reviews have inequalities as their primary focus. We found descriptive evidence of the differential impact of climate change on children in social groups by relative advantage versus disadvantage within countries and in LMICs compared with HICs; however, quantitative evidence comparing advantaged and disadvantaged children was limited.

Our findings suggest that although inequalities in child health due to climate change are recognized, the literature remains descriptive. For example, Benevolenza and DeRigne [21] describe the greater impact on the health of low-income households of flooding and infrastructure destruction resulting from hurricanes compared with high-income households. However, as the authors acknowledge, none of the studies they reviewed included quantitative evidence of the differential impact. Data on direct consequences of the flooding caused by hurricanes, such as differential hospital admission rates for diarrheal disease by household income among children exposed to the flooding, would provide a better measure of the extent to which hurricanes exacerbate child health inequality.

Understanding the mechanisms by which climate change may exacerbate child health inequalities is key to interventions to minimize its effects. Identification of specific pathways from climate change to child health inequalities using Bennett and Friel’s approach [42] is likely to inform interventions to modify amplification of inequalities by climate change. The urgent need for interventions to mitigate the effects of climate change is illustrated by the recent IPCC report [11] and echoed in the editorials published in 200 medical journals calling for emergency action to limit global temperature increases, restore biodiversity, and protect health [43]. Our scoping review has identified the limited capacity of LMICs to mitigate the negative effects of climate change due to scarce structural economic and political resources and, as demanded by the medical journal editorials [43], enhanced interventions by high income countries will be necessary to promote and fund mitigation and adaptation in LMICs, including improving the resilience of health systems.

Our review finds that much of the literature examines child physical health and infectious diseases, with little focus on child mental health and cognitive development. Most literature is focused on younger children, in particular less than 5 years of age. Children in these age groups are more vulnerable due to their incomplete physiological and cognitive development, but older children are more susceptible to climate change related anxiety.

The strengths of this scoping review of reviews include the development of a robust search strategy based on agreed protocol, as well as a structured screening and selection process based on consensus among the authors. In addition, the scoping review approach enabled us to reach a broad understanding of the state of knowledge and research activity in the area of child health inequalities and climate change. Following the recommendations of Arksey and O’Malley, we are confident that our findings can encourage a subsequent systematic review on this topic. Finally, our review had a lot of breadth and depth by assessing the impact of climate change globally.

Several limitations should be taken into account. The scoping review methodology precludes assessment of study quality in order to avoid focus on a hierarchy of evidence at the expense of breadth of evidence in an under-researched field. Establishing causal associations between climate change and inequalities in children’s health was not possible from existing evidence. The impact of climate change is dynamic with various exposures acting over long periods of time and few of the included reviews accounted for these aspects, except for those that emphasized “indirect” mechanisms. However, in the studies analyzed, a consistent, plausible relationship has been presented.

The review has identified knowledge gaps, including limited quantitative evidence of the impact of climate change across socioeconomic groups and between countries, limited examination and understanding of the mechanisms by which climate change exacerbates child health inequalities and minimal focus on the effect of climate change on child health inequalities for older children and on mental health. A program of research to address these gaps will require the measurement of the impact of climate change on child health across socioeconomic groups in defined populations in different settings and between countries and subnational regions. Existing socio-demographic surveys, such as the Demographic and Health Surveys (DHS) (Available online: https://dhsprogram.com/ (accessed on 8 October 2021)) and other national and regional surveys, could be modified to include relevant data.

Data quality must be considered to improve the validity of results. While maintaining policy and research focus on the most vulnerable children in poor and low resourced families and children in LMICs, it must also be recognized that more children are becoming increasingly vulnerable through various climate patterns. The implications of the relationship of climate change to social determinants of health for policy and research should be explored further. The interactions between social determinants of health and climate change are considered as indirect effects in some reviews [24,25,27,32] and climate change has been characterized as a social determinant of health [44]. Further work should explore how climate change acts both as a social determinant of health and an amplifier of other social determinants to increase child health inequalities, as suggested by Bennett and Friel [42].

## 5. Conclusions

The findings of the scoping review of reviews allow us to provide answers to the research questions we set out to address. There was agreement across the reviews that children, particularly poor children, and those in LMICs, are vulnerable to climate change; however, the current state of knowledge of the impact of climate change on child health inequalities is weak. Despite general acknowledgement in the included reviews of the likelihood that climate change will exacerbate child health inequalities, the reported evidence was marginal with little in-depth examination of this issue. There was a paucity of quantitative data comparing children across social groups and countries to support the evidence for the impact of climate change on child health inequalities and mechanisms by which climate change generates child health inequalities warrant further exploration. Our findings indicate the need for an enhanced program of research to address the knowledge gaps and provide evidence for effective interventions to mitigate climate change’s impact on child health inequalities. Further investigation of geographical and intergenerational inequality is also warranted.

## Figures and Tables

**Table 1:** Inclusion and exclusion criteria.

**Inclusion Criteria:**
Reviews, published to present, reviewing the literature on the relationship of climate change and its consequences and inequality in health outcomes among children aged 0–18 years. Reviews must report on the effect of climate change and its consequences on one or more health outcomes in different social groups and/or between high and low/middle income countries or geographical locations
**Exclusion Criteria:**
Reviews of literature on the general effects of climate change on child health without addressing the differential effect on social groups, different countries or geographical locations Reviews of literature on health outcomes among children and adults which do not report child health outcomes separately Reviews of literature on the effects of climate change on disadvantaged populations without reference to health effects on children aged 0–18 years

**Table 2:** Systematic reviews.

Authors	Journal	Research Question/Objective	Age of Study Population(s)	Context	Types of Climate Change	Child Health Outcomes	Dimension of Child Health Inequity	Summary of Review Findings Reinequalities and Type of Evidence (Quantitative and/ or Descriptive)
Benevolenza MA and DeRigne 2018 [21]	J Human Behavior Soc Env	Evaluate and summarize the research in peer-reviewed literature that pertains to climate change and natural disasters acting as destabilizing forces hampering the mental and physical health of vulnerable populations	0–18 years	US	Weather events; hurricanes; increase in annual numbers and intensity	Emotional and cognitive health, depression and anxiety	Within country— low v. high-income households	Low-income households at increased risk of maternal stress and child anxiety due to natural disasters compared with high-income households. Descriptive evidence only.
Phalkey RK, et al. 2015 [22]	PNAS	Assess the scientific evidence base for the impact of climate change on childhood undernutrition (particularly stunting) in subsistence farmers in low- and middle-income countries.	0–5 years	LMICs	Weather events, e.g., rainfall, extreme weather events (floods/droughts), seasonality, and temperature	Childhood undernutrition (stunting)	Within country— Low household income, low education Geographic—semi-arid conditions	Current evidence is limited but suggests a significant link between weather variables and stunting at household level in LMICs. Agricultural, socioeconomic, and demographic factors at the household and individual level play a substantial role in mediating the nutritional impacts of climate changes. Descriptive evidence only
Chersich et al. 2020 [23]	BMJ	Assess whether exposure to high temperatures in pregnancy is associated with increased risk for preterm birth, low birth weight, and stillbirth.	Perinatal	Global *	Increased temperatures, heat waves	Preterm birth, birth weight, and stillbirths	Within country: women in low SES, low educated groups. Between country—LMICs v. HICs	Associations between temp and outcomes were largest among women in lower SES groups, e.g., women in Korean study with both low education levels and low socioeconomic status had a 1.1-fold increased hazard ratio of preterm birth for each quartile increase in temperature, considerably higher than that in other women in the study. Some evidence suggested that pregnant women in low and middle income countries were vulnerable to heat exposure throughout pregnancy, whereas vulnerability among women in high income countries was largely confined to the last weeks of pregnancy. Descriptive and quantitative evidence.
Lieber et al. 2020 [24]	Global Public Health	Summarize evidence on relationship between climate change and malnutrition	0–18 years	Global *	Droughts, flooding and climate variability (CC proxies)	Malnutrition (wasting, stunting, or underweight)	Within country—low v. high maternal education; low v. high SES	Pooled estimates for wasting (OR 1.46 (95%CI 1.05,2.04)) and underweight (OR 1.46 (95%CI1.01,2.11) prevalence associated with drought Factors moderating impact of drought/flooding on malnutrition: 4 included studies found that higher levels of maternal education were protective against the development of malnutrition in children. and 5 studies found poorest families were most vulnerable to malnutrition. Descriptive and quantitative evidence

* Global = LMICs and HICs.

**Table 3:** Technical and commissioned reports.

Authors	Journal	Research Question/Objective	Age of Study Population(s)	Context	Types of Climate Change	Child Health Outcomes	Dimension of Child Health Inequity	Summary of Review Findings Reinequalities and Type of Evidence (Quantitative and/ or Descriptive)
Costello A, et al. 2009 [13]	The Lancet	Define climate change, define changes to patterns of illnesses that are affected by climate change.	0–18 years	Global *	Rising temperatures	Malnutrition, diarrhoea, infectious diseases, respiratory diseases, vector-borne diseases, deaths.	Within country—Poor children in urban areas, those in traditional societies and children of subsistence farmers Between country— LMICs v. HICs Geographic—coastal populations	Climate change will have its greatest effect on those who have the least access to the world’s resources and who have contributed least to its cause. Without mitigation and adaptation, it will increase health inequity especially through negative effects on the social determinants of health in the poorest communities. Descriptive and quantitative evidence
Ahdoot S, and Pacheco. 2015 [25]	Pediatrics	To educate pediatricians on the current knowledge of climate change and its effects on children’s health.	0–18 years	Global *	Rising temperatures	Heat-related mortality and morbidity including preterm birth; asthma and respiratory disease; infectious diseases; death and injury due to extreme weather events; malnutrition; diarrhoea	Within country—poorest households Between country— LMICs v, HICs	Children in the world’s poorest countries, where the disease burden is already disproportionately high, are most affected by climate change. Studies have projected a 7% to 20% increase in the number of malnourished children globally because of climate change primarily in LMICs. Descriptive and quantitative evidence
Assembly of First Nations (AFN) 2008 [26]	AFN Reports	Describe the vulnerability of FN children to environmental hazards including climate change	0–18 years	Canada	Rising temperatures	Infant mortality, respiratory infections, skin conditions, diarrhoea, mental health problems	Within country—First Nation children	Poverty, poor housing, over-crowding among first nation families Descriptive evidence

* Global = LMICs and HICs.

**Table 4:** Narrative reviews and opinion pieces.

Authors	Journal	Research Question/Objective	Age of Study Population(S)	Context	Types of Climate Change	Child Health Outcomes	Dimension of Child Health Inequity	Summary of Review Findings Reinequalities and Type of Evidence (Quantitative and/or Descriptive)
Clemens et al. 2020 [27]	European Child and Adolescent Psychiatry	Summarize evidence on direct and indirect pathways of climate change on child and adolescent mental health	0–18 years	Global *	Warming and natural disasters	Mental health, post-traumatic stress disorder	Within country—Adversity. Low income and disadvantaged families Between countries—low HDI V. high HDI Geographic—low lying areas	Mental health consequences of climate change will particularly affect children and adolescents who are already disadvantaged—those with low or no social support by families and peers, those from families with low socio-economic status. Socioeconomic factors such as, the human development index of the country are relevant pointing towards more severe effects in poverty on internal or external disorder or distress Descriptive evidence
Ebi and Paulson, 2007. [28]	Pediatr Clin N Am	Review the key issues related to climate change, then reviews climate-sensitive health determinants and outcomes in the context of children’s health, considers intergenerational equity issues	0–18 years	Global *	Rising temperatures; changes in global precipitation patterns, rising sea levels, and increases in the frequency and intensity of some extreme weather events	Mortality from heat events, infectious disease (e.g., Lyme disease), malnutrition, respiratory illnesses	Within country—Wealth and income distribution as key determinants of health impact of climate change Between country—LMICs v. HICs	Particular vulnerability of children (vs adults) to the adverse effects of climate change interact with poverty, race and class. Also introduces concept of ‘intergenerational inequity’, where children are considered to be a particularly disadvantaged population, not only on account of their present age-related vulnerability, but also because the higher likelihood of experiencing severe effects of climate change in the future. Descriptive evidence
Goldhagen JL, et al. 2019 [29]	The Lancet Child and Adolesc	Present a global agenda for child health and wellbeing as a blueprint for the practice of pediatrics and child health in the domains of clinical care, systems development, and policy formulation.	0–18 years	Global *	Climate change is increasing the frequency and intensity of extreme weather events,	Malaria, dengue, lepto-spirosis, and leishmaniosis; children’s stress, anxiety, depression, and post-traumatic stress disorder; Diarrhea, stunting, vector-borne disease	Between and within country, climate change as one of determinants of child health, basics human needs, and children’s rights	A disproportionate effect of climate change on the rights of specific groups of vulnerable children, including displaced children, children living in poverty, indigenous children, and children with developmental disabilities. Descriptive evidence
Kistin et al. 2010 [30]	Arch Dis Child	To evaluate how the rise in temperature, precipitation, droughts, floods, glacier melt and sea levels resulting from human-induced climate change is affecting the quantity, quality and flow of water resources worldwide and impacting child health through dangerous effects on water supply and sanitation, food production and human migration.	0–18 years	Global *	Floods, storms, drought and extreme weather events	Water related illnesses - malnutrition, diarrhea,	Between country— LMICs vs. HICs	Climate change estimate to cause approximately 5.5 million of disability adjusted life years in 2000, and predictions of an increase of 16% of malnutrition, 5% diarrhea and 17% of malaria by 2030. The risk will potentially be highest on the poorest and more vulnerable populations particularly among children Descriptive evidence
Levy and Patz, 2015. [31]	Ann Global Health.	Present overview of climate change manifestation and populations who will be most affected	0–18 years; general population	Global *	Heat waves, heavy precipitation events, intensity and duration of droughts, intense tropical cyclone, sea level	Heat-related disorders, vector-borne diseases, foodborne and waterborne diseases, respiratory and allergic disorders, malnutrition, collective violence, and mental health problems.	Within country—poor and marginalized in poor countries. Minority status Between country— HICs vs. LMICs Geographic—low altitude	Climate change disproportionately affects poor countries and poor people within these countries. Socioeconomic, demographic, health-related, geographic, and other risk factors make subgroups within populations more vulnerable to the adverse health effects of climate change. Adverse health effects caused by climate change likely to be heavily concentrated in low-income populations at low latitudes Descriptive evidence
McMichael 2014 [32]	Children	Provide overview of climate change manifestation and populations that will be most affected	0–18 years	Global *	Heat waves, heavy precipitation events, intensity and duration of droughts, intense tropical cyclone, sea level	Under-nutrition and stunting; diarrheal, parasitic, vector-borne and other infectious diseases; and allergic respiratory disorders [3,4]. Social and emotional development	Between country— LMICs v. HICs - initially LMICs most affected but increasingly effects will be felt across the world	Children in lower-income countries, especially in tropical areas, will suffer the most from climate change due to further amplification of health risks by persistent poverty, crowded living, lack of access to clean water, poor sanitation and inadequate healthcare systems Includes a summary of direct risks, less direct (secondary risks) and tertiary (diffuse) health consequences Descriptive evidence
Parkes M, et al. 2010 [33]	Int Public Health J.	Review key social determinants of health for Indigenous children and ultimately argue for a broader SDoH framework for Indigenous children.	0–18 years	Canada	Warming, loss of permafrost, degradation of habitats and species	Ecological and socio-cultural determinants of health of First Nations children. Specific outcomes not stated	Within country— Indigenous status with associated poverty, disadvantage, Geographic—vulnerability in their homelands	Indigenous children have poor health outcomes and weaker material infrastructure than non-Indigenous children; argue also that full wellbeing of Indigenous children is more broadly defined Descriptive evidence
Patz JA, et al. 2007 [34]	Eco Health	Provide overview of climate change manifestation and populations who will be most affected	0–18 years; general population; focus on under 5 child population	Global *	Undefined; rising temperatures	Malaria, malnutrition diarrhea	Between countries— LMICs vs. HICs	Existing health disparities for many people already struggling with poverty, malnutrition, and the effects of natural disasters will be exacerbated by climate change. Countries producing fewer emissions at higher risk from climate change. Example of malaria-endemic countries Descriptive evidence
Philipsborn RP and Chan, 2018. [35]	Pediatrics	Overview of climate change manifestation and populations who will be most affected	0–18 years	Global *	Warming, rising sea levels, increasing natural disasters, air pollution, desertification	Heat stress, malnutrition, diarrhea, vector-borne diseases, allergies	Within country— Poor vs. non-poor. Between country— LMICs vs. HICs	Children in resource-limited settings - households, countries - most affected by respiratory-related illness. These children also most vulnerable to droughts and flooding—magnifying existing disparities in social determinants of health Descriptive evidence
Rylander C, et al. 2013 [36]	Global Health	Review how climate change will increase the risk of infant and maternal mortality, birth complications, and poorer reproductive health, especially in tropical, developing countries.	Pregnant women, the developing fetus, and young children	LMICs	Global warming and extreme weather events	Infant mortality, preterm birth, low birth weight, malnutrition and stunting, diarrhea, malaria,	Between country—LMICs v. HICs; tropical most vulnerable	Climate change will have a substantial impact on the health and survival of the next generation among already challenged populations through impact of malnutrition on maternal health and pregnancy outcomes Descriptive evidence
Sheffield and Landrigan, [37]	Env Health Perspect	Review the projected impacts of climate change on children’s health, the pathways involved in these effects, and prevention strategies.	Fetus and 0–18 years	Global *	Warming, extreme weather events, rising sea levels, air pollution	Vector-borne diseases such as malaria and dengue; increased diarrheal and respiratory disease; increased morbidity and mortality from extreme weather; changed exposures to toxic chemicals; worsened poverty; food and physical insecurity; and threats to human habitation. Heat-related health effects for which research is emerging include diminished school performance, increased rates of pregnancy complications, and renal effects.	Within country—Poor, disadvantaged children Between countries— LMICs V. HICs	The health impacts of global climate change are expected to be widespread, geographically variable, and profoundly influenced by preexisting social and economic disparities. Stark variation in these outcomes is evident by geographic region and socioeconomic status, and these impacts will exacerbate health disparities Increased morbidity and mortality, vector-borne diseases, exposure to toxic chemical, threats to human habitation. Heat-related health effects and its relationship with school performance, increased rates of pregnancy complications, and renal effects. Descriptive evidence
Anderko et al. 2020 [38]	Pediatric research	Provide an overview of research exploring the impact of climate change on children’s health impacts, as well as provide recommendations for pediatric research moving forward.	Fetal and perinatal periods and 0–18 years	Global *	Warming, extreme weather events, rising sea levels, air pollution (CO_2_)	Asthma, allergies, vector-borne diseases, malnutrition, low birth weight, post-traumatic stress	Between countries— LMICs vs. HICs	Children in low-income countries that already experience a higher burden of disease and limited capability to adapt are affected even more by climate change. They are at high risk of vector-borne disease, malnutrition, worse perinatal outcomes, mental health, decreased school attendance Descriptive evidence
Wooldridge G and Murthy S. 2020 [39]	Frontiers in Pediatrics	Explore impact of climate change on pediatric critical care (PCC) and focusing on the health care sector’s impact on CC	0–18 years	Global *	Warming, extreme weather events	Critical illness related to heat stress, vector-borne disease, diarrhea, malnutrition, pneumonia	Between countries— LMICs vs. HICs	Climate change will increase burden of pediatric critical illness and disruption to health care systems and LMICs least able to cope. Descriptive evidence
Olson and Metz 2020 [40]	Faculty Reviews	Explore relationship between prenatal maternal stress (PNMS) and paternal stress, allostatic load, and the degradation of the environment on individuals.	Fetal, perinatal and 0–18 years	Global *	Warming, air and land pollution, extreme weather events, rising sea levels, ocean acidification	Preterm birth, low birth weight, mental health problems	Within country—Poor and indigenous newborns and children. Between countries— LMICs vs. HICs	Climate crisis as a health threat multiplier - amplifies the health inequities of the most at-risk populations and individuals. It accelerates the increase in allostatic load of those at risk leading to increased pre-term-birth, and developmental, and mental health problems Descriptive evidence
Davies GI, et al. 2014 [41]	Int J Env Res Public Health	Characterize the impact of weather events in Cambodia	0–18 years; general population	Cambodia	Weather events, especially floods, droughts and typhoons; Climate change is predicted to increase the frequency and intensity of such events.	Water-borne diseases, primarily diarrheal disease (i.e., viral and bacterial gastroenteritis, dysentery, cholera and other manifestations of gastrointestinal infections)	Within country— Poverty, lower level of education Geographic— Populations in low-lying areas	Pre-existing vulnerabilities and low adaptive capacity of the population relate to widespread poverty, poor health and malnutrition; settlements in flood-prone areas; reliance on agriculture for food security and income; low education levels; inadequate warning systems; and resource, governance and public health limitations Descriptive and quantitative evidence
Bennett and Friel 2014 [42]	Children	To address the amplification of existing child health inequities by climate change	0–18 years	Global *	Global heating, extreme weather events, changing precipitation patterns	Heat stress, vector-borne diseases and undernutrition	Within country—poorest and socially-disadvantaged Between country—LMICs v. HICs	The burden of climate change-related ill-health will fall heavily on the world’s poorest and socially-disadvantaged children, who already have poor survival rates and low life expectancies due to issues including poverty, endemic disease, undernutrition, inadequate living conditions and socio-economic disadvantage. Descriptive evidence

* Global = LMICs and HICs.

## Data Availability

Not applicable.

## Supplementary Materials

The following are available online at https://www.mdpi.com/article/10.3390/ijerph182010896/s1, Tables S1 and S2: Search Terms from Scoping Review.

## References

[1] WHO Commission on Social Determinants of Health (2008). Closing the Gap in a Generation: Health Equity Through Action on the Social Determinants of Health. Final Report of the Commission on Social Determinants of Health.

[2] Spencer N., Raman S., O’Hare B., Tamburlini G. (2019). Addressing inequities in child health and development: Towards social justice. BMJ Paediatr. Open.

[3] UNICEF (2015). Progress for Children No.11. Beyond Averages: Learning from the MDGs.

[4] da Silva I.C.M., França G.V., Barros A.J., Amouzou A., Krasevec J., Victora C. (2018). Socioeconomic Inequalities Persist Despite Declining Stunting Prevalence in Low- and Middle-Income Countries. J. Nutr..

[5] Yaya S., Bishwajit G. (2019). Burden of Acute Respiratory Infections Among Under-Five Children in Relation to Household Wealth and Socioeconomic Status in Bangladesh. Trop. Med. Infect. Dis..

[6] Pinzón-Rondón A.M., Zárate-Ardila C., Hoyos-Martínez A., Ruiz-Sternberg Á.M., Velez-Van-Meerbeke A. (2015). Country characteristics and acute diarrhea in children from developing nations: A multilevel study. BMC Public Health.

[7] Njau J.D., Stephenson R., Menon M., Kachur S.P., A McFarland D. (2013). Exploring the impact of targeted distribution of free bed nets on households bed net ownership, socio-economic disparities and childhood malaria infection rates: Analysis of national malaria survey data from three sub-Saharan Africa countries. Malar. J..

[8] Hajat A., Hsia C., O’Neill M.S. (2015). Socioeconomic Disparities and Air Pollution Exposure: A Global Review. Curr. Environ. Health Rep..

[9] Pollard C.M., Booth S. (2019). Food Insecurity and Hunger in Rich Countries—It Is Time for Action against Inequality. Int. J. Environ. Res. Public Health.

[10] Harker L. (2006). Chance of a Lifetime: Impact of Bad Housing on Children’s Lives.

[11] Masson-Delmotte V., Zhai P., Pirani A., Connors S.L., Péan C., Berger S., Caud N., Chen Y., Goldfarb L., Gomis M.I. (2021). Climate Change 2021: The Physical Science Basis. Contribution of Working Group I to the Sixth Assessment Report of the Intergovernmental Panel on Climate Change.

[12] McMichael A.J., Campbell-Lendrum D.H., Corvalan C.F., Ebi K.L., Githeko A., Scheraga J.D., Woodward A. (2003). Climate Change and Human Health: Risks and Responses.

[13] Costello A., Abbas M., Allen A., Ball S., Bell S., Bellamy R., Friel S., Groce N., Johnson A., Kett M. (2009). Managing the health effects of climate change: Lancet and University College London Institute for Global Health Commission. Lancet.

[14] Helldén D., Andersson C., Nilsson M., Ebi K.L., Friberg P., Alfvén T. (2021). Climate change and child health: A scoping review and an expanded conceptual framework. Lancet Planet. Health.

[15] Arksey H., O’Malley L. (2005). Scoping studies: Towards a methodological framework. Int. J. Soc. Res. Methodol..

[16] Levac D., Colquhoun H., O’Brien K.K. (2010). Scoping studies: Advancing the methodology. Implement. Sci..

[17] Munn Z., Peters M.D.J., Stern C., Tufanaru C., McArthur A., Aromataris E. (2018). Systematic review or scoping review? Guidance for authors when choosing between a systematic or scoping review approach. BMC Med. Res. Methodol..

[18] Fakoya O.A., McCorry N.K., Donnelly M. (2020). Loneliness and social isolation interventions for older adults: A scoping review of reviews. BMC Public Health.

[19] Enns J.E., Holmqvist M., Wener P., Halas G., Rothney J., Schultz A., Goertzen L., Katz A. (2016). Mapping interventions that promote mental health in the general population: A scoping review of reviews. Prev. Med..

[20] OHCHR Convention on the Rights of the Child 1990. https://www.ohchr.org/en/professionalinterest/pages/crc.aspx.

[21] Benevolenza M.A., DeRigne L. (2019). The impact of climate change and natural disasters on vulnerable populations: A systematic review of literature. J. Hum. Behav. Soc. Environ..

[22] Phalkey R.K., Aranda-Jan C., Marx S., Höfle B., Sauerborn R. (2015). Systematic review of current efforts to quantify the impacts of climate change on undernutrition. Proc. Natl. Acad. Sci. USA.

[23] Chersich M.F., Pham M.D., Areal A., Haghighi M.M., Manyuchi A., Swift C.P., Wernecke B., Robinson M., Hetem R., Boeckmann M. (2020). Associations between high temperatures in pregnancy and risk of preterm birth, low birth weight, and stillbirths: Systematic review and meta-analysis. BMJ.

[24] Lieber M., Chin-Hong P., Kelly K., Dandu M., Weiser S.D. (2020). A systematic review and meta-analysis assessing the impact of droughts, flooding, and climate variability on malnutrition. Glob. Public Health.

[25] Ahdoot S., Pacheco S.E., The Council on Environmental Health Global (2015). Climate Change and Children’s Health. Pediatrics.

[26] Assembly of First Nations The Health of First Nations Children n.d.:28. https://www.afn.ca/uploads/files/rp-discussion_paper_re_childrens_health_and_the_environment.pdf.

[27] Clemens V., Von Hirschhausen E., Fegert J.M. (2020). Report of the intergovernmental panel on climate change: Implications for the mental health policy of children and adolescents in Europe—A scoping review. Eur. Child Adolesc. Psychiatry.

[28] Ebi K.L., Paulson J.A. (2007). Climate Change and Children. Pediatr. Clin. North Am..

[29] Goldhagen J.L., Shenoda S., Oberg C., Mercer R., Kadir A., Raman S., Waterston T., Spencer N.J. (2020). Rights, justice, and equity: A global agenda for child health and wellbeing. Lancet Child Adolesc. Health.

[30] Kistin E.J., Fogarty J., Pokrasso R.S., McCally M., McCornick P.G. (2010). Climate change, water resources and child health. Arch. Dis. Child..

[31] Levy B.S., Patz J.A. (2015). Climate Change, Human Rights, and Social Justice. Ann. Glob. Health.

[32] McMichael A.J. (2014). Climate Change and Children: Health Risks of Abatement Inaction, Health Gains from Action. Children.

[33] Parkes M., de Leeuw S., Greenwood M. (2010). Warming Up to the Embodied Context of First Nations Health: A Critical Intervention into and Analysis of Health and Climate Change Research. Int. Public Health J..

[34] Patz J.A., Gibbs H.K., Foley J.A., Rogers J.V., Smith K.R. (2007). Climate Change and Global Health: Quantifying a Growing Ethical Crisis. EcoHealth.

[35] Philipsborn R.P., Chan K. (2018). Climate Change and Global Child Health. Pediatrics.

[36] Rylander C., Odland J., Øyvind, Sandanger T.M. (2013). Climate change and the potential effects on maternal and pregnancy outcomes: An assessment of the most vulnerable–The mother, fetus, and newborn child. Glob. Health Action.

[37] Sheffield P.E., Landrigan P.J. (2011). Global Climate Change and Children’s Health: Threats and Strategies for Prevention. Environ. Health Perspect..

[38] Anderko L., Chalupka S., Du M., Hauptman M. (2019). Climate changes reproductive and children’s health: A review of risks, exposures, and impacts. Pediatr. Res..

[39] Wooldridge G., Murthy S. (2020). Pediatric Critical Care and the Climate Emergency: Our Responsibilities and a Call for Change. Front. Pediatr..

[40] Olson D.M., Metz G.A. (2020). Climate change is a major stressor causing poor pregnancy outcomes and child development. F1000Research.

[41] Davies G.I., McIver L., Kim Y., Hashizume M., Iddings S., Chan V. (2015). Water-Borne Diseases and Extreme Weather Events in Cambodia: Review of Impacts and Implications of Climate Change. Int. J. Environ. Res. Public Health.

[42] Bennett C.M., Friel S. (2014). Impacts of Climate Change on Inequities in Child Health. Children.

[43] Atwoli L., Baqui A.H., Benfield T., Bosurgi R., Godlee F., Hancocks S., Horton R., Laybourn-Langton L., Monteiro C.A., Norman I. (2021). Call for emergency action to limit global temperature increases, restore biodiversity, and protect health. J. Health Popul. Nutr..

[44] Ragavan M.I., Marcil L., Garg A. (2020). Climate Change as a Social Determinant of Health. Pediatrics.

