# Supplementary material for: Climate Change and Child Health Inequality: A Review of Reviews
Source: Int J Environ Res Public Health. 2021 Oct 17;18(20):10896. doi: 10.3390/ijerph182010896 (PMC8535343; doi:10.3390/ijerph182010896)
Supplement: Supplementary file 1 [file ijerph-18-10896-s001.zip › ijerph-1408429-supplementary.pdf]

Table S1. Summary of Selected Reviews.

Authors	Year	Title	Journal	Research Question/Objective	Type of review	Age of study population(s)	Context	Types of climate change	Child health outcomes
Benevolenza MA, et al.	2018	The impact of climate change and natural disasters on vulnerable populations: A systematic review of literature	J Human Behavior Soc Env	Evaluate and summarize the research in peer-reviewed literature that pertains to climate change and natural disasters acting as destabilizing forces hampering the mental and physical health of vulnerable populations	Systematic review	0-18	US	Weather events; hurricanes; increase in annual numbers and intensity	Emotional and cognitive health, depression and anxiety
Phalkey RK, et al.	2015	Systematic review of current efforts to quantify the impacts of climate change on undernutrition	PNAS	Assess the scientific evidence base for the impact of climate change on childhood undernutrition (particularly stunting) in subsistence farmers in low- and middle-income countries.	Systematic review	<5 years of age	LMICs	Weather events, e.g., rainfall, extreme weather events (floods/droughts), seasonality, and temperature	Childhood undernutrition (stunting)
Ahdoot S,et al.	2015	Global climate change and children's health	Pediatrics	Educate pediatricians on the current knowledge of climate change and its effects on children's health.	Review undefined	0-18	Global	Rising temperatures	Heat-related mortality & morbidity including preterm birth; asthma & respiratory disease; infectious diseases; death & injury due to extreme weather events; malnutrition; diarrhoea
Costello A, et al.	2009	Managing the health effects of climate change	The Lancet	Define climate change, define changes to patterns of illnesses that are affected by climate change.	Review undefined	0-18	Global	Rising temperatures	Malnutrition, diarrhoea, infectious diseases, respiratory diseases, vector-borne diseases, deaths.

Assembly of First Nations (AFN)	2008	The Health of First Nations Children and the Environment: Discussion Paper	AFN Reports	Describe the vulnerability of FN children to environmental hazards including climate change	Review undefined	0-18	Canada	Rising temperatures	Infant mortality, respiratory infections, skin conditions, diarrhoea, mental health problems
Davies GI, et al.	2014	Water-borne diseases and extreme weather events in Cambodia: review of impacts and implications of climate change	Int J Env Res Public Health	Characterize the impact of weather events in Cambodia	Non-systematic reviews and opinion articles	0-18; general population	Cambodia	Weather events, especially floods, droughts and typhoons; Climate change is predicted to increase the frequency and intensity of such events.	Water-borne diseases, primarily diarrhoeal disease (i.e., viral and bacterial gastroenteritis, dysentery, cholera and other manifestations of gastrointestinal infections)
Ebi, et al.	2007	Climate change and children	Pediatr Clin N Am	Review the key issues related to climate change, then reviews climate-sensitive health determinants and outcomes in the context of children's health, considers intergenerational equity issues	Review undefined	0-18	Global	Rising temperatures; changes in global precipitation patterns, rising sea levels, and increases in the frequency and intensity of some extreme weather events	Mortality from heat events, infectious disease (e.g., Lyme disease), malnutrtion, respiratory illnesses
Goldhagen JL, et al.	2019	Rights, justice, and equity: a global agenda for child health and wellbeing	The Lancet Child & Adolesc	Present a global agenda for child health and wellbeing as a blueprint for the practice of paediatrics and child health in the domains of clinical care, systems development, and policy formulation.	Review undefined	0-18	Global	Climate change is increasing the frequency and intensity of extreme weather events,	Malaria, dengue, lepto-spirosis, and leishmaniosis; children's stress, anxiety, depression, and post-traumatic stress disorder; Diarrhoea, stunting, vector-borne disease

Kistin et al.	2010	Climate change, water resources and child health	Arch Dis Child	evaluate how the rise in temperature, precipitation, droughts, floods, glacier melt and sea levels resulting from human-induced climate change is affecting the quantity, quality and flow of water resources worldwide and impacting child health through dangerous effects on water supply and sanitation, food production and human migration.	Review undefined	0-18	Global	Floods, storms, drought & extreme weather events	Water related illnesses - malnutrition, diarrhea,
Levy, et al.	2015	Climate change, human rights, and social justice	Ann Global Health.	Present overview of climate change manifestation and populations who will be most affected	Review undefined	0-18; general population	Global	Heat waves, heavy precipitation events, intensity and duration of droughts, intense tropical cycloe, sea level	Heat-related disorders, vector-borne diseases, foodborne and waterborne diseases, respiratory and allergic disorders, malnutrition, collective violence, and mental health problems.
McMichael	2014	Climate Change and Children: Health Risks of Abatement inaction, Health Gains from Action	Children	Provide overview of climate change manifestation and populations that will be most affected	Review undefined	0-18	Global	Heat waves, heavy precipitation events, intensity and duration of droughts, intense tropical cycloe, sea level	Under-nutrition and stunting; diarrhoeal, parasitic, vector-borne and other infectious diseases; and allergic respiratory disorders [3,4]. Social and emotional development
Parkes M, et al.	2010	Warming Up to the Embodied Context of First Nations Health: A Critical Intervention into and Analysis of Health and Climate Change Research	Int Public Health J.	Review key social determinants of health for Indigenous children and ultimately argue for a broader SDoH framework for Indigenous children.	Review undefined	0-18	Canada	Warming, loss of permafrost, degradation of habitats & species	Ecological & socio-cultural determinants of health of First Nations children. Specific outcomes not stated

Patz JA, et al.	2007	Climate change and global health: Quantifying a growing ethical crisis	Eco Health	Provide oveview of climate change manifestation and populations who will be most affected	Review undefined	0-18; general population; focus on under 5 child population	Global	Undefined; rising temperatures	Malaria, malnutrition diarrhoea
Philipsborn RP, et al.	2018	Climate change and global child health	Pediatrics	Overview of climate change manifestation and populations who will be most affected	Review undefined	0-18	Global	Warming, rising sea levels, increasing natural disasters, air pollution, desertific-ation	Heat stress, malnutrition, diarrhoea, vector-borne diseases, allergies
Rylander C, et al.	2013	Climate change and the potential effects on maternal and pregnancy outcomes: an assessment of the most vulnerable - the mother, fetus, and newborn child	Global Health	Review how climate change will increase the risk of infant and maternal mortality, birth complications, and poorer reproductive health, especially in tropical, developing countries.	Review undefined	Pregnant women, the developing fetus, and young children	Tropical and developing countries	Global warming & extreme weather events	Infant mortality, preterm birth, low birth weight, malnutrition & stunting, diarrhoea, malaria,
Sheffield, et al.	2011	Global Climate Change and Children's Health: Threats and Strategies for Prevention	Env Health Perspect	Review the projected impacts of climate change on children's health, the pathways involved in these effects, and prevention strategies.	Review undefined	Fetus & 0-18	Global	Warming, extreme weather events, rising sea levels, air pollution	Vector-borne diseases such as malaria and dengue; increased diarrheal and respiratory disease; increased morbidity and mortality from extreme weather; changed exposures to toxic chemicals; worsened poverty; food and physical insecurity; and threats to human habitation. Heat-related health effects for which research is emerging include diminished school performance, increased rates of pregnancy

									complications, and renal effects.
Anderko et al.	2020	Climate changes reproductive and children's health: a review of risks, exposures and impacts	Pediatric research	Provide an overview of research exploring the impact of climate change on children's health impacts, as well as provide recommendations for pediatric research moving forward.	Review undefined	Fetal & perinatal periods & 0-18	Global	Warming, extreme weather events, rising sea levels, air pollution (CO2)	Asthma, allergies, vector-borns diseases, malnutrition, low birth weight, post-traumatic stress
Chersich et al.	2020	Associations between high temperatures in pregnancy and risk of preterm birth, low birth weight, and stillbirths: systematic review and meta-analysis	BMJ	Assess whether exposure to high temperatures in pregnancy is associated with increased risk for preterm birth, low birth weight, and stillbirth.	Systematic review & m/analysis	Perinatal	Global	Increased temperatures, heat waves	Preterm birth, birth weight, and stillbirths
Clemens et al.	2020	Report of the intergovernmental panel on climate change: implications for the mental health policy of children and adolescents in Europe—a scoping review	European Child & Adolescent Psychiatry	Summarize evidence on direct and indirect pathways of climate change on child and adolescent mental health	Review undefined	0-18	Global	Warming & natural disasters	Mental health, post-traumatic stress disorder

Lieber et al.	2020	A systematic review and meta-analysis assessing the impact of droughts, flooding, and climate variability on malnutrition	Global Public Health	Summarize evidence on relationship between climate change and malnutrition	Systematic review & meta-analysis	0-18	Global	Droughts, flooding and climate variability (CC proxies)	Malnutrition (wasting, stunting, or underweight)
Wooldridge G and Murthy S	2020	Pediatric critical care and the climate emergency: Our responsibilities and a call for change	Frontiers in Pediatrics	Explore impact of climate change on pediatric critical care (PCC) and focusing on the health care sector's impact on CC (vicious circle?)	Review	0-18	Global	Warming, extreme weather events	Critical illness related to heat stress, vector-borne disease, diarrhoea, malnutrition, pneumonia
Olson and Metz	2020	Climate change is a major stressor causing poor pregnancy outcomes and child development	Faculty Reviews	Explore relationship between prenatal maternal stress (PNMS) and paternal stress, allostatic load, and the degradation of the environment on individuals.	Review undefined	Fetal, perinatal & 0-18	Global	Warming, air & land pollution, extreme weather events, rising sea levels, ocean acidific-ation	Preterm birth, low birth weight, mental health problems

Table S2. Search Terms from Scoping Review.

1. Medline

1. exp Climate Change/
2. Greenhouse Effect/
3. exp Hot Temperature/
4. exp Natural Disasters/

5. (avalanche* or climate change* or cyclonic storm* or drought* or greenhouse effect* or extreme heat or heat wave* or hot temperature* or flood* or global warming or landslide* or natural disaster* or sea level rise* or tidal wave* or tornado* or weather or wildfire*).ti,ab,kf.
6. or/1-5
7. Adolescent Health/
8. Child Health/
9. Child Welfare/
10. Child Mortality/
11. Infant Health/
12. Infant Welfare/
13. exp Infant Mortality/
14. Adolescent/
15. exp Child/
16. exp Infant/
17. (adolescen* or boy* or child* or girl* or infant* or juvenile* or minor* or neonatal* or newborn* or pediatric* or paediatric* or preschool* or toddler* or teen* or youth* or young*).ti,ab,kf.
18. or/7-17
19. Health Status/
20. Health Status Disparities/
21. Social Determinants of Health/
22. exp Socioeconomic Factors/
23. ((economic* or health or social* or socio*) adj3 (condition* or determinant* or disadvant* or disparit* or effect* or factor* or inequit* or ineqalit* or status)).ti,ab,kf.
24. (poverty or underdevelop*).ti,ab,kf.
25. or/19-24
26. Mental Health/
27. Anxiety/
28. Depression/
29. Adaptation, Psychological/
30. Resilience, Psychological/

31. Psychological Trauma/
32. Stress, Psychological/
33. ((anxiety or coping or depression or emotional distress or mental health) adj7 (adolescen* or boy* or child* or girl* or infant* or juvenile* or minor* or neonatal* or newborn* or pediatric* or paediatric* or preschool* or toddler* or teen* or youth* or young*)).ti,ab,kf.
34. ((psych* or mental) adj3 (adaptation or effect* or resilience or stress or trauma or wellbeing or well-being) adj7 (adolescen* or boy* or child* or girl* or infant* or juvenile* or minor* or neonatal* or newborn* or pediatric* or paediatric* or preschool* or toddler* or teen* or youth* or young*)).ti,ab,kf.
35. or/26-34
36. 25 or 35
37. 6 and 18 and 36
38. 37 not (animals not humans).sh.
39. Review.pt.
40. Systematic Review.pt.
41. review*.ti.
42. or/39-41
43. 38 and 42

2. Embase

('climate change'/exp OR 'greenhouse effect'/de OR 'high temperature'/de OR avalanche*:ti,ab,kw OR 'climate change*':ti,ab,kw OR 'cyclonic storm*':ti,ab,kw OR drought*:ti,ab,kw OR 'greenhouse effect*':ti,ab,kw OR 'extreme heat':ti,ab,kw OR 'heat wave*':ti,ab,kw OR 'hot temperature*':ti,ab,kw OR flood*:ti,ab,kw OR 'global warming':ti,ab,kw OR landslide*:ti,ab,kw OR 'natural disaster*':ti,ab,kw OR 'sea level rise*':ti,ab,kw OR 'tidal wave*':ti,ab,kw OR tornado*:ti,ab,kw OR weather:ti,ab,kw OR wildfire*:ti,ab,kw)

AND

('adolescent health'/de OR 'child health'/de OR 'child welfare'/exp OR 'childhood mortality'/de OR 'infant welfare'/de OR 'infant mortality'/de OR 'adolescent'/de OR 'child'/exp OR 'infant'/exp OR adolescen*:ti,ab,kw OR boy*:ti,ab,kw OR child*:ti,ab,kw OR girl*:ti,ab,kw OR infant*:ti,ab,kw OR juvenile*:ti,ab,kw OR minor*:ti,ab,kw OR neonatal*:ti,ab,kw OR newborn*:ti,ab,kw OR pediatric*:ti,ab,kw OR paediatric*:ti,ab,kw OR preschool*:ti,ab,kw OR toddler*:ti,ab,kw OR teen*:ti,ab,kw OR youth*:ti,ab,kw OR young*:ti,ab,kw)

AND

('health status'/de OR 'health disparity'/de OR 'social determinants of health'/de OR 'socioeconomics'/exp OR (((economic* OR health OR social* OR socio*) NEAR/3 (condition* OR determinant* OR disadvant* OR disparit* OR effect* OR factor* OR inequit* OR ineqalit* OR status)):ti,ab,kw) OR poverty:ti,ab,kw OR underdevelop*:ti,ab,kw OR 'mental health'/exp OR 'anxiety'/de OR 'depression'/de OR 'adolescent depression'/de OR 'coping behavior'/de OR 'psychological resilience'/de OR 'psychotrauma'/de OR 'mental stress'/de OR (((anxiety OR coping OR depression OR 'emotional distress' OR 'mental health') NEAR/7 (adolescen* OR boy* OR child* OR girl* OR infant* OR juvenile* OR minor* OR neonatal* OR newborn*

OR pediatric* OR paediatric* OR preschool* OR toddler* OR teen* OR youth* OR young*)):ti,ab,kw) OR (((psych* OR mental) NEAR/3 (adaptation OR effect* OR resilience OR stress OR trauma OR wellbeing OR 'well being') NEAR/7 (adolescen* OR boy* OR child* OR girl* OR infant* OR juvenile* OR minor* OR neonatal* OR newborn* OR pediatric* OR paediatric* OR preschool* OR toddler* OR teen* OR youth* OR young*)):ti,ab,kw))

NOT ([animals]/lim NOT [humans]/lim)

AND ([article]/lim OR [article in press]/lim OR [review]/lim)

AND [review]/lim

3. Web of Science Core Collection

#1 TOPIC: ((avalanche* or "climate change*" or "cyclonic storm*" or drought* or "greenhouse effect*" or "extreme heat" or "heat wave*" or "hot temperature*" or flood* or "global warming" or landslide* or "natural disaster*" or "sea level rise*" or "tidal wave*" or tornado* or weather or wildfire*))

AND

#2 TOPIC: ((adolescen* or boy* or child* or girl* or infant* or juvenile* or minor* or neonatal* or newborn* or pediatric* or paediatric* or preschool* or toddler* or teen* or youth* or young*))

AND

#3 TOPIC: (((economic* or health or social* or socio*) NEAR/3 (condition* or determinant* or disadvant* or disparit* or effect* or factor* or inequit* or ineqalit* or status))) OR TOPIC: ((poverty or underdevelop*)) OR TS=(((anxiety or coping or depression or "emotional distress or mental health") NEAR/7 (adolescen* or boy* or child* or girl* or infant* or juvenile* or minor* or neonatal* or newborn* or pediatric* or paediatric* or preschool* or toddler* or teen* or youth* or young*))) OR TS=((((psych* or mental) NEAR/3 (adaptation or effect* or resilience or stress or trauma or wellbeing or well-being)) NEAR/7 (adolescen* or boy* or child* or girl* or infant* or juvenile* or minor* or neonatal* or newborn* or pediatric* or paediatric* or preschool* or toddler* or teen* or youth* or young*)))

#4 #1 AND #2 AND #3 Refined by: DOCUMENT TYPES: (ARTICLE OR CORRECTION OR REVIEW OR EARLY ACCESS)

#5 #1 AND #2 AND #3 DOCUMENT TYPES: (REVIEW)

5. Psychinfo

1. exp climate change/
2. heat effects/
3. exp natural disasters/
4. (avalanche* or climate change* or cyclonic storm* or drought* or greenhouse effect* or extreme heat or heat wave* or hot temperature* or flood* or global warming or landslide* or natural disaster* or sea level rise* or tidal wave* or tornado* or weather or wildfire*).ti,ab,id.
5. or/1-4

6. adolescent health/
7. child welfare/
8. (adolescen* or boy* or child* or girl* or infant* or juvenile* or minor* or neonatal* or newborn* or pediatric* or paediatric* or preschool* or toddler* or teen* or youth* or young*).ti,ab,id.
9. or/6-8
10. health status/
11. health disparities/
12. exp socioeconomic status/
13. ((economic* or health or social* or socio*) adj5 (condition* or determinant* or disadvant* or disparit* or effect* or factor* or inequit* or ineqalit* or status)).ti,ab,id.
14. (poverty or underdevelop*).ti,ab,id.
15. or/10-14
16. exp mental health/
17. exp emotional states/
18. coping behavior/
19. "resilience (psychological)"/
20. emotional trauma/
21. psychological stress/
22. ((anxiety or coping or depression or emotional distress or mental health) adj7 (adolescen* or boy* or child* or girl* or infant* or juvenile* or minor* or neonatal* or newborn* or pediatric* or paediatric* or preschool* or toddler* or teen* or youth* or young*)).ti,ab,id.
23. ((psych* or mental) adj3 (adaptation or effect* or resilience or stress or trauma or wellbeing or well-being) adj7 (adolescen* or boy* or child* or girl* or infant* or juvenile* or minor* or neonatal* or newborn* or pediatric* or paediatric* or preschool* or toddler* or teen* or youth* or young*)).ti,ab,id.
24. or/16-23
25. 15 or 24
26. 5 and 9 and 25
27. 26 not (animals not humans).sh.
28. review*.ti.
29. 26 and 28
